# Application of an oral health-related quality of life questionnaire in primary care patients with orofacial pain and temporomandibular disorders

**DOI:** 10.4317/medoral.19061

**Published:** 2013-10-13

**Authors:** Antonio Blanco-Aguilera, Antonio Blanco-Hungría, Lourdes Biedma-Velázquez, Rafael Serrano-del-Rosal, Laura González-López, Elena Blanco-Aguilera, Rafael Segura-Saint-Gerons

**Affiliations:** 1 Odontologist. MS in Orofacial Pain, Temporomandibular Disorders and Orthodontics USP-CEU, Madrid, Spain; 2 MD. Stomatologist, Andalusian Healthcare Service. Researcher, Maimónides Institute for Biomedical Research of Cordoba, Spain. Assistant Professor, Department of Medicine, University of Cordoba, Spain; 3 Sociologist. Senior Research Technician, Institute for Advanced Social Studies, Spanish National Research Council (IESA-CSIC); 4 PhD in Sociology. CSIC Research Scientist, Institute for Advanced Social Studies, Spanish National Research Council (IESA-CSIC); 5 Odontologist. MS in Pediatric Dentistry. University of Seville, Spain; 6 Odontologist. MS student in Oral Medicine, Surgery and Implantology, University of Santiago de Compostela, Spain; 7 MD. Stomatologist, Andalusian Healthcare Service. Assistant Professor, Department of Medical and Surgical Specialties, University of Cordoba, Spain

## Abstract

Objectives: To examine whether patients who report orofacial pain (OP) and temporomandibular disorders (TMD) have a poorer perception of their oral health-related quality of life and, if so, to what extent, and to analyze the association between oral health perception, sociodemographic variables and reported pain duration. Study Design: 407 patients treated at the OP and TMD units in the Healthcare District of Cordoba, Spain, diagnosed following the standard criteria accepted by the scientific community – the Research Diagnostic Criteria for Temporomandibular Disorders (RDC/TMD) – were administered the Spanish version of the Oral Health Impact Profile questionnaire (OHIP-14). Bivariate and logistic regression analyses were performed to determine the degree of association between the patients’ OHIP-14 score and pain duration, pain intensity, and various sociodemographic variables. Results: The observed distribution was 89.4% women and 10.6% men. The mean OHIP-14 score was 20.57 ± 10.73 (mean ± standard deviation). A significant association (p<0.05) was found for gender, age, marital status, chronic pain grade, self-perceived oral health status and pain duration. Conclusions: The analysis of self-perceived oral health status in patients with OP and TMD, as measured by the OHIP-14, showed that oral health is perceived more negatively by women. Moreover, a one-point increase in the Chronic Pain Grade indicator increases the OHIP-14 indicator by 4.6 points, while chronic pain, defined as pain suffered by patients for one year or more, increases the OHIP-14 indicator by 3.2 points.

** Key words:**Orofacial pain, temporomandibular disorders, Oral Health Impact Profile, sociodemographic variables, primary care, Research Diagnostic Criteria for Temporomandibular Disorders (RDC/TMD).

## Introduction

Temporomandibular disorders (TMD) is a collective term used to refer to a number of clinical conditions that involve the masticatory musculature and/or temporomandibular (TM) joints and associated structures, or both (1). The main symptom of such disorders is localized pain in the orofacial region, which is defined by the International Association for the Study of Pain (IASP) as “an unpleasant sensory and emotional experience associated with actual or potential tissue damage, or described in terms of such damage”. In addition to pain, patients might also present other symptoms such as joint sounds (clicking and crepitus), which can, in turn, be related to alterations or limitations in mandibular dynamics.

This multi-etiological approach covers a wide range of etiological causes, including macro-traumas or micro-traumas in the para-functions (i.e. bruxism), skeletal and occlusal alterations (which according to the literature have decreased in this type of disorder in recent years), systemic factors (arthritis, alterations in collagen metabolism, etc.), masticatory and cervical muscle hyperactivity, alterations of the collagen matrix in temporomandibular joint (TMJ) cartilage, hormonal factors especially in women, and genetic factors, primarily catechol-o-methyltransferase (COMT).

Psychological factors can also contribute to the etiological causes of TMD, particularly physical symptoms which may or may not be associated with the painful symptoms that accompany situations of emotional stress, resulting in increased excitability of the head and neck muscles. Depression is observed especially among elderly patients with limited masticatory function due to intra-artricular disorders, particularly osteoarthritis.

According to two studies by Poveda et al., there is a high prevalence of TMD among the Spanish population. In their second study, the authors found that 31.4% and 18.1% of the control group presented disc displacement with reduction (DDWR) and myofascial pain (MFP), respectively (2), compared to 44.8% and 35.2% of patients in the first study (3). Due to its high preva-lence, it is important that TMD be assessed in specific primary care units using a standardized clinical examination and diagnostic protocol.

The Research Diagnostic Criteria for Temporomandibular Disorders (RDC/TMD) is a dual-axis assessment tool used to obtain a clinical and psychological diagnosis. Axis II of the RDC/TMD, which is consistent with a biopsychosocial health model, assesses a number of items, including pain intensity, graded chronic pain and psychological variables such as depression, anxiety and physical symptoms.

In the last two decades, the RDC/TMD has proven to be a highly valid and reliable instrument, which has enabled the standardization of clinical subtypes (4), as well as providing grading scales for pain intensity, disability and psychological discomfort in patients with TMD (5). However, the RDC/TMD lacked a qualitative approach to self perceived oral health due to the scarcity of items related to this concept. This limitation was overcome by administering additional questionnaires relating to patients’ self-perception of oral health-related quality of life that focus on the importance of assessing functional status, health status and quality of life as related concepts within a wide spectrum (6).

Some of the standard questionnaires used for this purpose are the Geriatric Oral Health Assessment Index (GOHAI), the Oral Health-Related Quality of Life (OHQoL), and the Oral Health Impact Profile (OHIP-49). The OHIP-49 was developed and vali-dated by Slade and Spencer (7) and contains 49 questions that capture the seven dimensions of Locker’s theoretical model of oral health (8). The Spanish version of the OHIP-49 was validated by Lopez (9) in a group of 9,163 students, confirming its validity and reliability for clinical use. However, it was later found that although the OHIP-49 was effective, it was difficult to administer. This prompted the original author (Slade) to later develop a shortened version, the Oral Health Impact Profile-14 (OHIP-14) (10), which largely coincides with the previous version, but lacks its limitations. The OHIP has been validated in several languages, including Chinese (11), Sinhalese (12), Hebrew (13), Swedish (14), Italian (15), German (16), Greek (17), and Portuguese (18), among others. In Spain, Montero-Martin et al. (19) validated the OHIP-14 in a sample of Spanish adults, demonstrating that the questionnaire was a valid, accurate, and reliable tool for measuring oral health-related quality of life.

The OHIP-14 is widely used in various branches of dentistry such as caries, periodontal disease, oral medicine, prosthetics, surgery, and others. Its application in patients with signs and symptoms of orofacial pain (OP) and TMD has also been widely described by several authors (6) (20), who have concluded that the OHIP-14 may play a role in predicting clinical deterioration in these patients (21).

The aim of this paper is to quantify the self-perceived health of patients with OP and TMD in order to subsequently analyze the correlation between self-perceived health and a series of variables. Furthermore, we attempt to empirically assess the relationship between self-perceived oral health and the chronification of TMD in patients seen by the Andalusian Healthcare Service primary care units.

## Material and Methods

-Study group and design

We conducted a cross-sectional epidemiological study in the Healthcare District of Cordoba, Spain, in a population of 415 potential patients with signs and symptoms of OP and TMD. All the patients were informed about the examination and were asked to complete a questionnaire in accordance with the norms and guidelines previously established for the study by the ethics committee of the Reina Sofía Teaching Hospital in Cordoba, Spain. Eight patients who refused to undergo the examination or complete the questionnaire were excluded from the study. From January 2011 to November 2012, the remaining 407 patients were examined by a specialist with 27 years of experience in OP and TMD, and completed the OP and TMD primary care unit questionnaire. All of the participants in the study were aged 16 and over (the minimum age required in the Autonomous Region of Andalusia for signing consent forms) and reported at least one of the following signs and symptoms: pain in the jaw or TMJs, restricted or limited range of motion when opening or closing the mouth or lateral excursions of the jaw, and joint sounds (with or without pain).

In addition to refusal to participate in the study or sign the informed consent form, the following exclusion criteria were also applied: systemic rheumatic disease (with the exception of fibromyalgia and rheumatoid arthritis); neurological or autoimmune diseases; patients who had undergone TMJ surgery or head and neck radiation treatment; patients who had suffered head and neck trauma two months prior to the study; pregnant patients; patients treated with narcotic analgesics, muscle relaxants or corticosteroids whose treatment could not be suspended one week prior to the study; patients who had been treated with antidepressants and NSAIDs at least three days prior to the study; and drug-dependent patients.

We used the Spanish version of the RDC/TMD materials originally designed by Dworkin et al. (22) to diagnose OP and TMD disorders. This version provides a clear explanation of each step of the procedure, as well as instructions to ensure that the data are standardized and comparable for research purposes.

In accordance with the RDC/TMD, patients with OP and TMD are assessed based on their clinical history and a physical examination using clinical decision algorithms in order to obtain a clinical classification. The RDC/TMD also includes sociodemographic and psychological variables.

For the qualitative analysis of self-perceived oral health in patients with OP and TMD, we used the Spanish version of the OHIP-14 questionnaire. The OHIP-14 comprises 14 items ( Table 1); each with five response categories corresponding to a 5-point Likert scale where 0 is “*never*” suffered problems or pain, and 4 is “*very often*”. The OHIP-14 score is obtained directly from the sum of the results of each of its 14 items (OHIP-14 = ∑ v1+ v2 +… + v14).

**Table 1:**
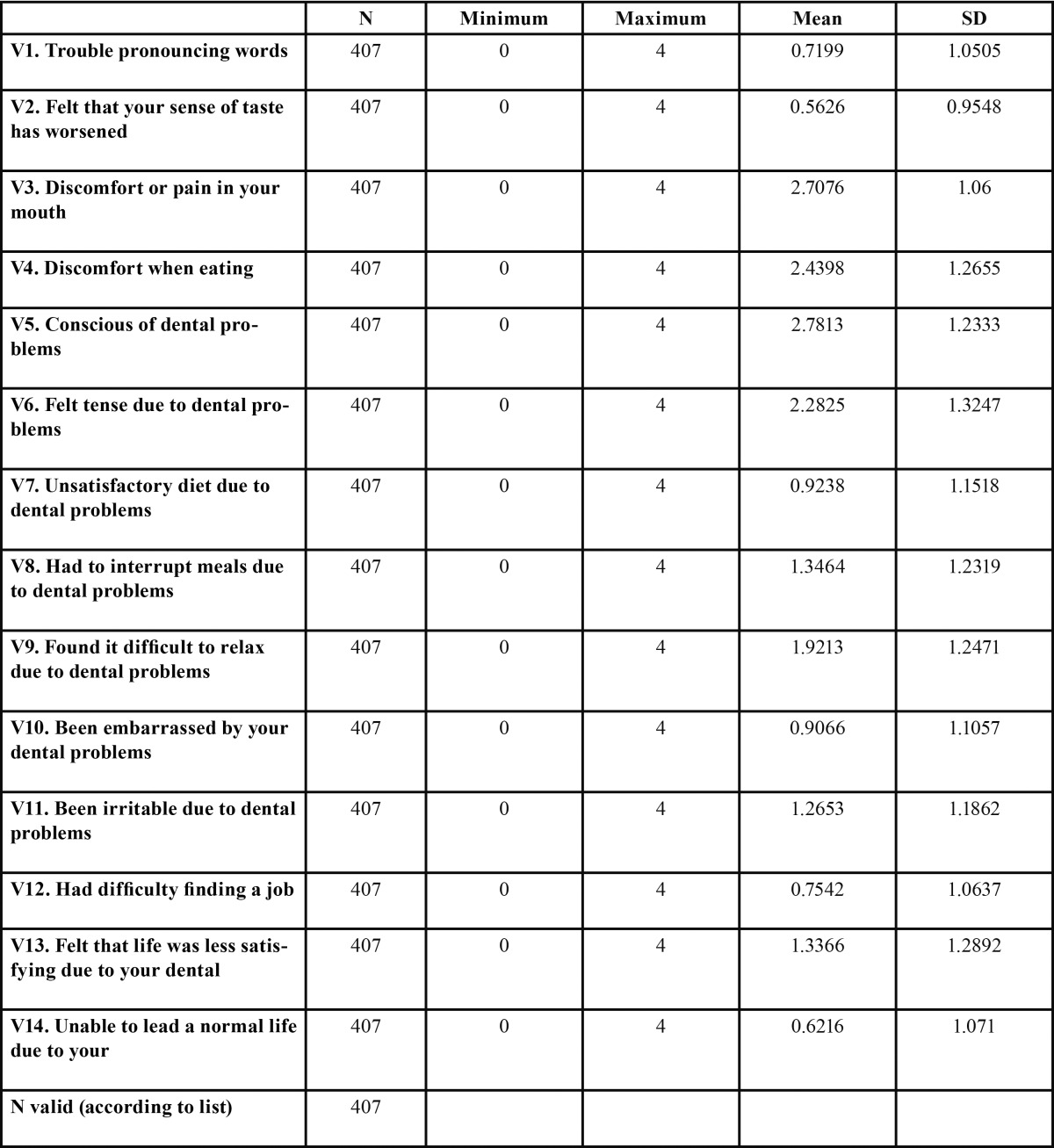
Descriptive statistics of the OHIP-14 items.

After administering the OHIP-14, we asked the participants to answer a final question on self-perceived oral health status, which had three possible responses: “*poor*”, “*fair*”, and “*good*”.

We also calculated the “chronic pain grade” as is habitually described in the literature (22). Chronic pain grade was obtained by two indicators: A) “Pain Intensity”, which was determined using visual analogue scales (VAS) (current pain intensity, maximum pain intensity and average pain intensity/3); and B) “Level of disability”, which was determined by quantifying the number of disability days and the focus of the disability. This was obtained from the sum of the VAS scores (how the disability in question affects patients’ daily, recreational and work activities).

**Figure 1:**
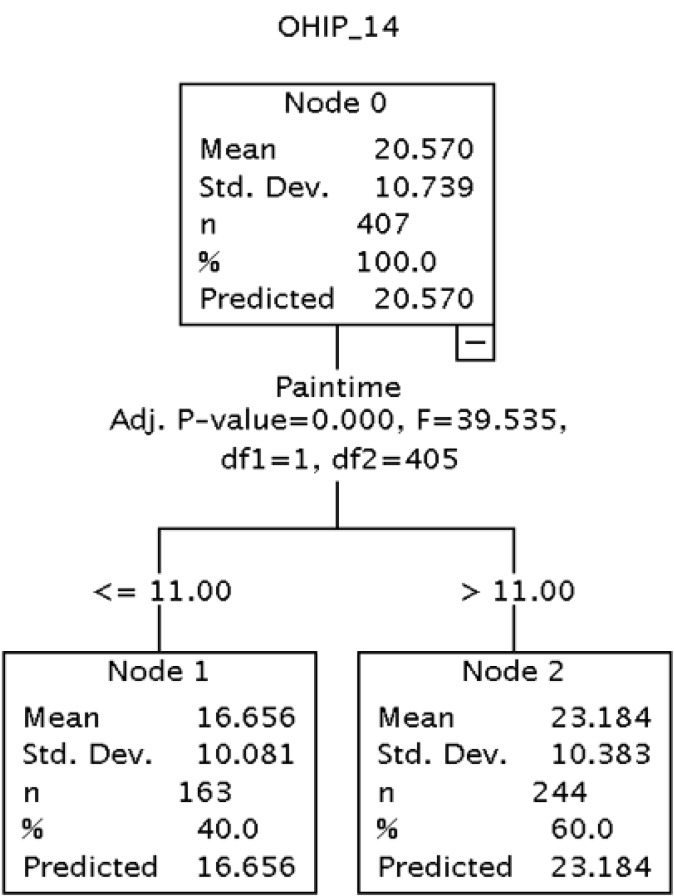
Hierarchical segmentation. Dependent variable: OHIP-14. Independent variable: pain duration measured in months.

It is also important to note that the variable pain duration used in the subsequent analyses was divided into two groups: one comprising patients who had suffered from pain for less than a year, and another composed of patients who had suffered from pain for one year or more. These groups were formed by means of a hierarchical segmentation analysis in which the dependent variable was the OHIP-14 score and the independent variable was pain duration measured in months. This analysis allowed us to statistically discriminate those groups which differed in relation to the dependent variable. It also permitted us to obtain an appropriate number of groups and establish the cutoff point taking into account only the statistical differences observed in the empirical evidence. The results are shown in figure 1.

-Statistical analysis

We first analyzed the relationship between the OHIP-14 scores and sociodemographic variables, oral health status, and pain duration. To do so, we performed a bivariate descriptive analysis. When the independent variables were categorical, we performed a contrast of means using Snedecor’s F-statistic. For the quantitative variables we performed a bilateral correlation analysis using Spearman’s rho and the Pearson correlation coefficient.

In the second phase, we performed a linear regression analysis using the OHIP-14 indicator as the dependent variable and the variables that were found to be statistically significant in the bivariate analysis as explanatory variables.

We tested for collinearity between the independent variables. To avoid this problem, we analyzed the partial correlations and the indicators of collinearity, as well as statistical tolerance and the variance inflation factor (VIF). By doing so, we were able to decrease the number of variables that were ultimately included in the model. This permitted us to obtain a result that was free from collinearity and fulfilled the principle of parsimony, according to which, all things being equal, when two results have the same outcome (in this case a similar R2) the simplest solution is more likely to be correct than the more complex solution. The data were analyzed using SPSS version 15.

## Results

The sample comprised 365 women (89.7%) with a mean age of 42.15 ± 14.63 (mean ± standard deviation), and 42 men (10.3%) with a mean age of 41.48 ± 17.28. Hence, the ratio of women to men was 8,4 with an age range of 16 to 83. The mean OHIP-14 score obtained was 20.57 ± 10.73 (95% CI , 19.5-21.61).

**Figure 2:**
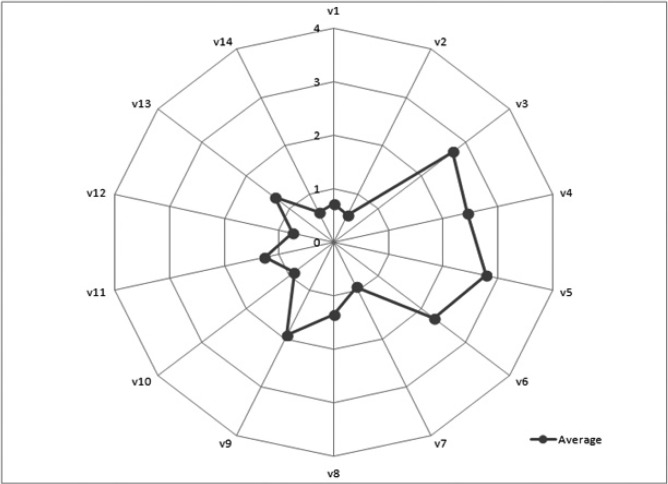
Values for all the OHIP-14 questions.

The distribution of the mean scores and standard deviations for each of the indicators are shown in  Table 1 and figure 2. As can be seen, some of the items show a higher mean score than others. For example, question 3, “Have you ever experienced discomfort or pain in your mouth?”; item 4, “Have you ever felt discomfort when eating certain types of food because of problems with your teeth, mouth or dentures?”; item 5, “Are you conscious of any problems you might have with your teeth, mouth or dentures?”; and finally, item 6 “Have you ever felt tense because of problems you have with your teeth, mouth or dentures?” The mean scores for these four items were higher than for the rest of the variables except for item 9, which asks: “Have you ever found it difficult to relax because of problems with your teeth, mouth or dentures?”, whose mean score is close to the others.

In contrast, item 1, “Have you ever had trouble pronouncing certain words because of problems with your teeth, mouth or dentures?”, as well as item 2, “Have you ever felt that your sense of taste has worsened because of problems with your teeth, mouth or dentures?”, and 14, “Have you ever felt incapable of leading a normal life because of problems with your teeth, mouth or dentures?”, show significantly lower scores than the rest of the variables in the questionnaire.

**Figure 3:**
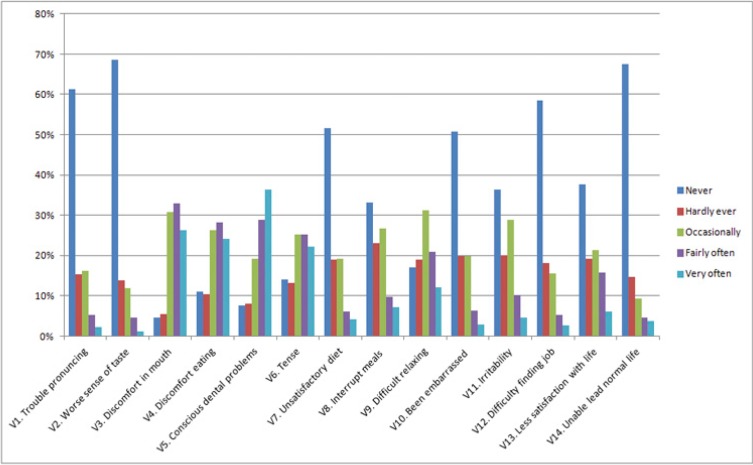
Values for all the OHIP-14 questions.

Figure 3 shows the distribution of the responses expressed as frequencies in a bar-chart for each of the items that comprise the OHIP-14 questionnaire. As can be seen, the response “never” is the modal score, except in the variables which ask directly about pain or oral discomfort. The most common response is “fairly often” and “very often”, with “never” and “hardly ever” being relatively infrequent responses among these patients.

 Table 2 shows the bivariate analysis between the OHIP-14 and the other variables included in the analysis. As can be observed, the most significant variables were gender, age groups, marital status, chronic pain grade, self-perceived oral health status, and pain duration.

**Table 2:**
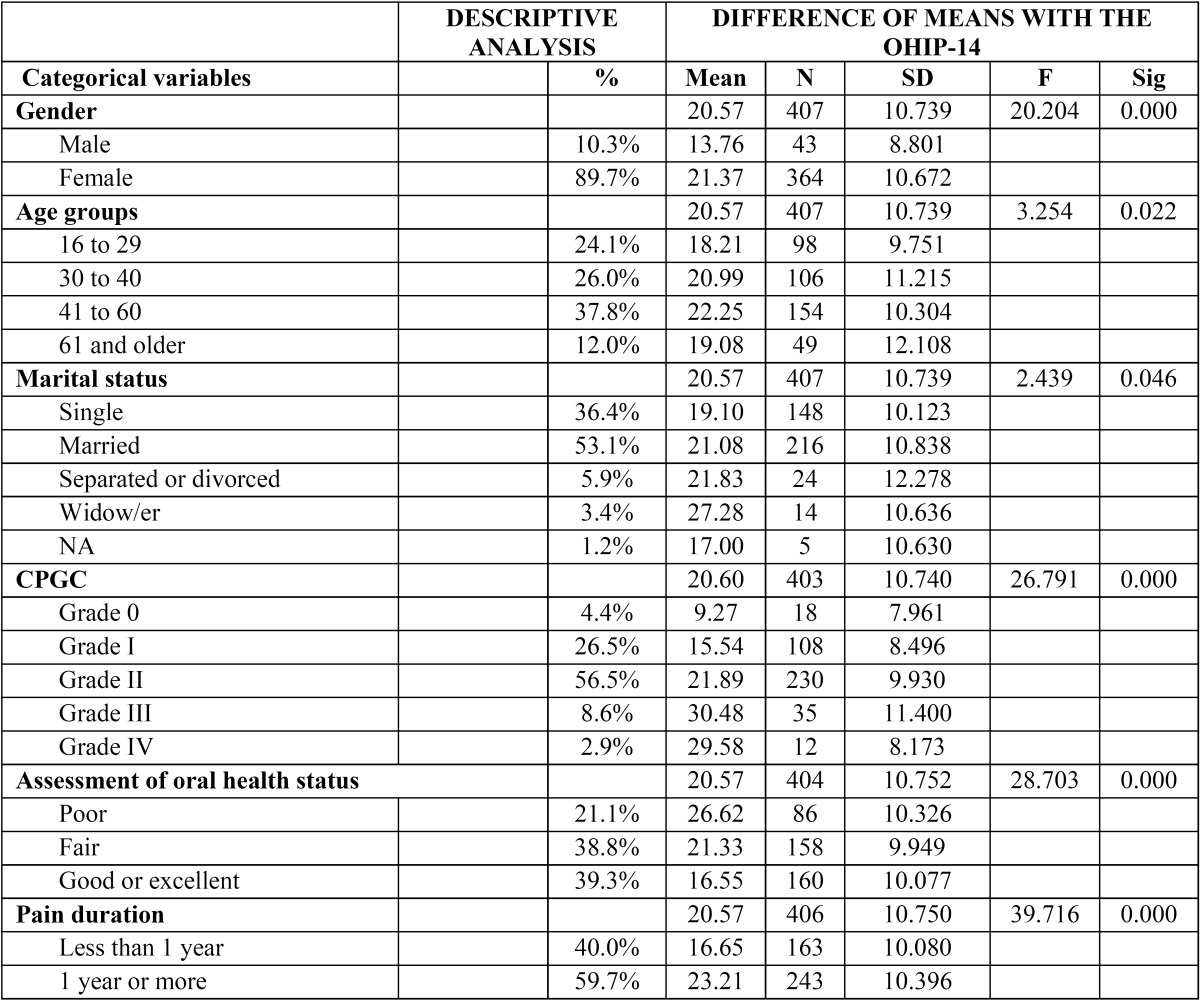
Descriptors of the sociodemographic, psychological and health variables and difference of means with the OHIP-14.

We also observed that women had higher OHIP-14 scores than men (21.37 vs. 13.79). As regards age groups, the highest number of patients was in the 41 to 60 age range, with the lowest number of patients in the 61 and over age range. A bivariate analysis was performed on the OHIP-14 score, obtaining a value of 0.022 for a significance level of p<0.05%.

As regards marital status, married patients comprise more than half of the sample, followed by single patients, while the remaining groups show practically residual values. The bivariate analysis of the OHIP-14 score showed a value of 0.046 (for p≤ 0.05), revealing that the distribution of the groups varies in relation to the OHIP-14 variable (21.08 for married patients compared to 19.10 for single patients).

As regards the chronic pain grade indicator, most patients reported high pain intensity with a low level of disability (Grade II), or low pain intensity without disability (Grade I), while less than 20% of the patients reported the other situations. The bivariate analysis of chronic pain grade compared to the OHIP-14 shows a high association between patients with high pain intensity without disability, and poor perception of oral health-related quality of life.

We also analyzed the association between the OHIP-14 score and self-perceived oral health status, finding as much as a 10-point difference in the OHIP-14 score between those who considered they had excellent oral health and those who rated their oral health as poor.

The last variable in the analysis was “pain duration”. This variable was included in the analysis in order to assess whether it had an impact on the OHIP-14 scores, i.e. whether patients who report more months of pain obtain different scores on the OHIP-14. This hypothesis was confirmed as patients who suffered from pain for more than eleven months obtained, on average, a score that was 6.56 points higher than the mean OHIP-14 score compared to the group who suffered pain for a shorter period of time.

 Table 3 shows the values obtained from a linear regression analysis using the OHIP-14 as the dependent variable and the variables that showed an association with the OHIP-14 score in the bivariate analysis as independent variables, i.e. sociodemographic variables, chronic pain grade and pain duration. We found that all the variables, with the exception of age groups and marital status, are significant in the regression model. Overall, the model is significant and has a high coefficient of determination (R2), which explains 31.2% of the OHIP-14 variance.

**Table 3:**
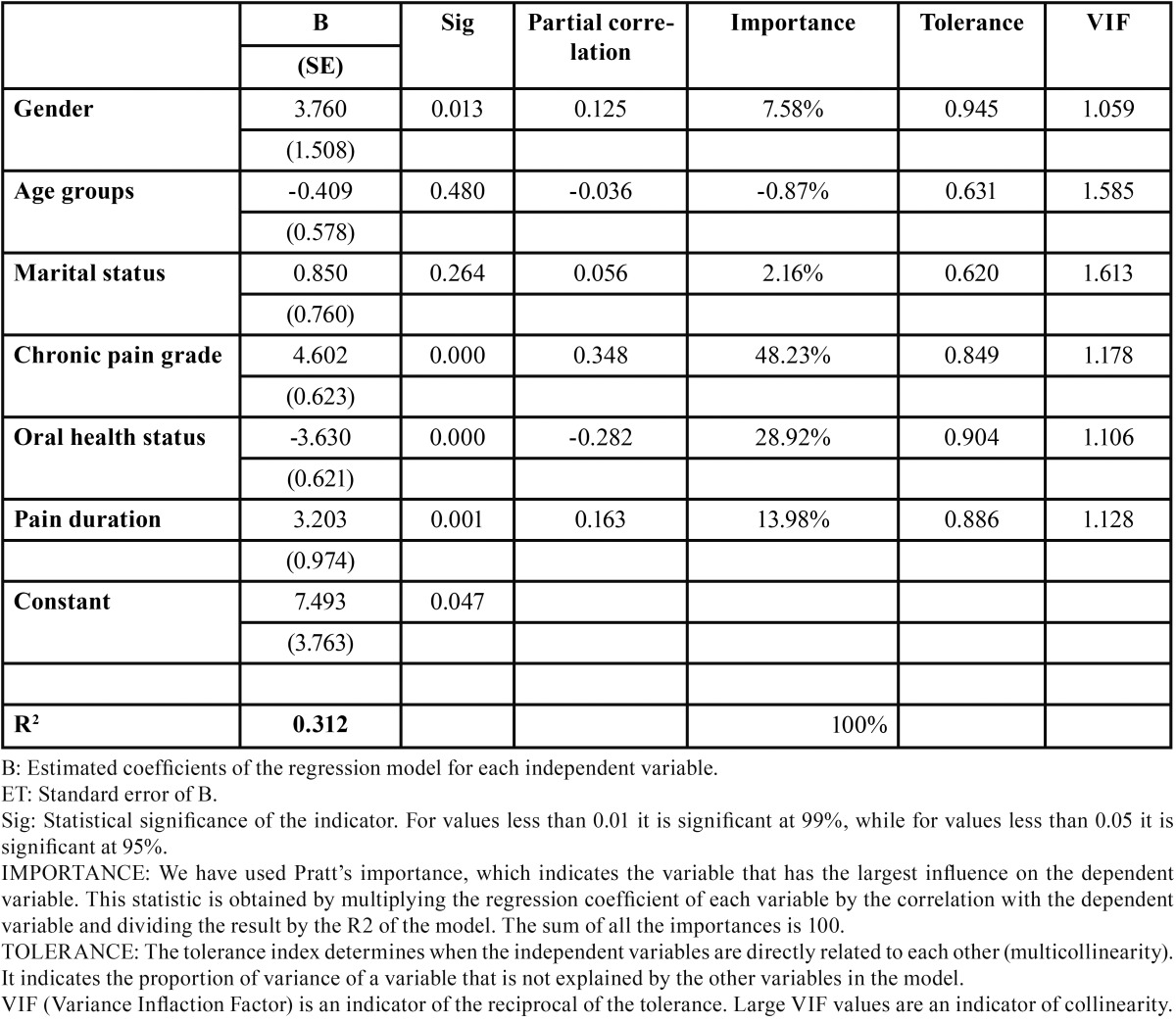
Regression analysis. Dependent variable: OHIP-14.

## Discussion

In recent years, self-perceived oral health-related quality of life questionnaires have been widely used in different dental specialties as well as in the general population in Spain and other countries. This has led to a better and more thorough understanding of people’s perception about oral health-related quality of life. However, the use of such questionnaires in patients seen in OP and TMD primary care units in Spain and their association with these disorders has not been analyzed and reported in the scientific community.

We observed higher mean OHIP-14 scores for OP and TMD patients than those reported by Montero et al. (23) in a sample of adults in Granada, Spain. In another article (19), however, the author reported significant differences in OHIP-14 scores between patients who perceived the need for dental treatment (95% CI, 10.4-13.2), and those who did not (95% CI, 6.7- 8.9). Moreover, as in our study, the author found higher mean scores for the items relating to physical pain and psychological discomfort than for the rest of the items on the OHIP-14 questionnaire.

The analysis of the association between perceived oral health-related quality of life in our patients by gender shows that the mean score for women with OP and TMD is 7.61 times higher than that for men. According to the regression model, a one-unit increase in gender, i.e. being female rather than male, increases the OHIP-14 score by almost 4 points, keeping all other variables in the regression model constant. These data are similar to those obtained by Rusanen et al. (24) in studies performed in patients with malocclusion and TMD, who concluded that women reported poorer oral health-related quality of life than men with malocclusion and TMD. As regards age groups, the OHIP-14 scores increase until 61 years of age when patients begin to have a better perception of their oral health-related quality of life, although the scores are always lower than those of adolescents and young adults. However, when introducing the scores in the regression model, they did not explain the changes observed in the OHIP-14 results. In our opinion, and from a purely hypothetical point of view, this could be attributed to the effect of two questions. The first is that self-perceived oral health should be compared at the intra-group level given that the groups comprise different strata of patients, i.e. older patients do not rate their health status in comparison to adolescents but with regard to the health status ascribed to their own peer group. It therefore seems reasonable to assume that patients over 60 compare themselves with the older age group in an aggregate and unconscious way, and thus report better self-perceived oral health. The second hypothesis, which is purely statistical in nature, is that this change is due to an effect produced by the sample given that the number of patients aged 61 and over account for almost half of all the other groups included in the study. In a similar vein, however, studies in groups of elderly patients by authors such as Stenman et al. (25) state that older patients report a poorer perception of their oral health-related quality of life, associating this phenomenon with denture-related problems and dry mouth syndrome, both of which limit mastication. Nonetheless, we believe that new studies focusing on elderly patients are needed in order to provide greater clarity and knowledge.

Although the variable marital status showed a degree of association in the chi-square bivariate analysis with p-values below 0.05, it was not found to be significant in the regression model. Although this might seem to contradict the results of Blanco et al. (26), who found a positive correlation between marital status and pain, their study analyzed pain intensity, while our study analyzes the OHIP-14 questionnaire; two dependent variables which do not measure the same thing. Chronic pain grade is one of the variables that shows a higher association with poorer perception of oral health-related quality of life. In our study, we found a 21-point difference for this variable between the mean scores of the groups who had a more positive perception of oral health-related quality of life (no pain groups) and patients who had a poorer perception of their oral health (high pain intensity and high disability). We observed that the mean score for chronic pain in the second group (high pain intensity and high disability) increases progressively until reaching a peak, when it falls only slightly. However, this group accounted for only a small portion of the total sample. The regression model shows that with each one-point increase in the chronic pain variable, the OHIP-14 score increases by 4.6 points, while the other variables included in the equation remain constant. Authors such as Miettinen et al. (27) evaluated the association between OHIP-14 prevalence and Axis II profile sub-scales. They observed that patients suffering from more severe chronic pain have a poorer perception of their oral health-related quality of life as well as higher levels of somatization and depression.

Furthermore, we observed that patients who rate their oral health status as poor also have a poorer perception of their oral health-related quality of life as measured by the OHIP-14, with the difference in mean scores decreasing by up to 5 percentage points in these patients. As regards self-perceived oral health status, a change in the assessment from 1 “poor” to 2 “fair” reduces the OHIP-14 score by 3.6 points, if all other variables remain constant. These results coincide consistently with those of other authors such as Montero and Bravo in a number of papers addressing this phenomenon (23,19).

Finally, as regards self-perceived oral health-related quality of life in terms of pain duration, where pain for “more than or less than 11 months” is taken as a cutoff point (not a hypothetical cutoff point but one that we observed empirically as explained above), we observed that patients who reported having suffered from pain for more than 11 months scored 3.2 points more in the OHIP-14 questionnaire than those who report having suffered from pain for less time, keeping all other variables constant.

In conclusion, all the variables, with the exception of age groups and marital status, are significant in the regression model. The overall model is significant and has a high coefficient of determination (R2), which explains 31.2% of the OHIP-14 variability. This indicates that the percentage of variance of the dependent variable can be explained by the independent variables, and accounts for 100% of the variability in the OHIP-14 (in this case 31.2%). As can be observed, chronic pain grade was the independent variable that most influences variability in the OHIP-14 (explaining 48.2% of the OHIP-14 variability), followed by self-perceived oral health status, which explains 28.9% of the variability; and pain duration, which explains 13.9% of the variability in the OHIP-14. Finally, the variable gender explains 7.6% of the variability in the OHIP-14.
